# Dietary Iron Intake in Pregnant Women in Europe: A Review of 24 Studies from 14 Countries in the Period 1991–2014

**DOI:** 10.1155/2020/7102190

**Published:** 2020-02-24

**Authors:** Nils Thorm Milman

**Affiliations:** Department of Clinical Biochemistry, Næstved Hospital, University College Zealand, DK-4700 Næstved, Denmark

## Abstract

**Objective:**

Assessment of dietary iron intake in pregnant women in Europe.

**Design:**

Review. *Setting*. Literature search of dietary surveys reporting the intake of dietary iron using the PubMed and Google Scholar databases covering the years 1990–2019.

**Subjects:**

Healthy pregnant women.

**Results:**

24 dietary surveys/studies in 14 European countries were included. Nine studies (38%) used Food Frequency Questionnaires, which yielded significantly higher iron intake than studies using Dietary Records. Results from Dietary Record studies in 11 countries showed that iron intake varied between 8.3–15.4 mg/day with an estimated “median” value of 10–11 mg/day. Spain, Bosnia, and Poland reported an intake of 8.3–10.1 mg/day, Croatia, England, Norway, and Finland an intake of 10.2–11.4 mg/day, and Germany, Portugal, Czech Republic, and Greece an intake of 12.2–15.4 mg/day. The recommended iron intake in the various countries varied from 14.8–30 mg/day. In all studies, 60–100% of the women had a dietary iron intake below the recommended intake.

**Conclusions:**

In Europe, the majority of pregnant women have a dietary iron intake, which is markedly below the recommended intake. This contributes to a low iron status in many pregnant women. Most guidelines do not advice routine iron supplements, while two guidelines (World Health Organization and Nordic Nutrition Recommendations) recommend routine iron supplementation during pregnancy. Within the European community, we need to reach consensus on the various guidelines and on the issue of iron supplementation. We should establish common European standardized dietary methods, uniform Dietary Reference Values, and uniform statistical methods in order to perform more reliable comparisons between studies in different countries.

## 1. Introduction

Body iron balance is a resultant of iron uptake vs. iron losses. In healthy humans, iron uptake is generated by gastrointestinal absorption of dietary iron [1]. In women of reproductive age, iron losses consist of obligatory or basal iron losses as well as physiological iron losses in association with menstruations [2, 3] and pregnancies [4, 5].

During pregnancy, there is a drastic physiologic increase in the need for uptake of iron compared to the nonpregnant period [5]. The need for absorbed iron increases from the 1st to the 3rd trimester with an average iron requirement in the entire gestation period of approximately 4.4 mg/day [5].

An appropriate iron homeostasis or iron status is crucial for a normal function of all cells, tissues, and organs in the body. Both iron deficiency (ID) and iron overload will affect body functions in negative ways and impair the quality of life as well as life expectancy [6, 7]. ID and iron deficiency anemia (IDA) during pregnancy has negative effects on the health status of the mother and predisposes to complications during gestation and at delivery [8] as well as to postpartum anemia [9]. Iron is important for the normal development of the organs of the fetus, especially for the brain [10]. Furthermore, ID will affect the newborn baby, causing premature delivery and low birthweight [8].

The World Health Organization's (WHO) report on the global prevalence of anemia [11] states that in the European region, among pregnant women 15–49 years of age, between 20.0–39.9% have anemia. The mean prevalence of anemia is 24.5% (95% confidence interval 17.8–33.8%). The predominant cause of anemia is ID [11].

The high physiological need for iron poses demands on the dietary intake and absorption of iron, which in turn are dependent on both the quantitative and qualitative dietary iron intake. In Europe, approximately 40% of women of reproductive age have small or absent body iron reserves, i.e., serum ferritin values <30 *μ*g/L [12]. The low iron status in these women is in part due to a low and inadequate dietary iron intake when hold against the recommended intake, as shown in a recent review article [13]. Among Danish women of reproductive age, only 20–30% have adequate body iron reserves, i.e., serum ferritin >60–70 *μ*g/L [14], making it possible for them to go through a pregnancy without taking iron supplements and without developing ID and IDA.

Among ethnic Danish pregnant women not taking supplemental iron, many develop ID and approximately 25% develop IDA [5, 14]. This indicates that dietary iron intake and iron absorption in the majority of pregnant women are inadequate and do not fulfill the normal physiological requirements.

We know that a large fraction of nonpregnant women of reproductive age in Europe have an inadequate dietary iron intake [13]. How is the situation when they become pregnant? Do they change their dietary habits and increase their intake of dietary iron? Or do they continue with their habitual prepregnancy diet?

The purpose of this paper is to provide a review of dietary surveys assessing dietary iron intake in pregnant women in Europe and to examine to which degree iron intake may fulfill the demands of the recommended intake.

## 2. Methods

Literature search was performed in PubMed using the MeSH Database terms (iron, dietary AND women, pregnant) and in Google Scholar using the terms “iron, dietary,” and “pregnancy” or “pregnant women.” The search yielded 1,020 articles from different parts of the world. European studies, performed in the period 1990–2019, which reported the dietary intake of micronutrients and iron *per se* in healthy pregnant women, were included in this review.

As shown in our previous paper on dietary intake in women of reproductive age in Europe [13], dietary surveys using the Food Frequency Questionnaire (FFQ) method overestimate the dietary iron intake [15]. Therefore, we decided *a priori* not to include studies using FFQs. However, during the literature search, it became evident that the total number of studies was small and that 9 out of the 24 identified studies (38%) used FFQs exclusively. In order to provide the reader with a more comprehensive overview of the available data, we subsequently decided to include the FFQ studies.

In the statistical interpretation of the results, it is important to consider the frequency distribution of dietary iron intake. If the distribution is normal, it is relatively simple to define and calculate inadequate intake using parametric statistics (arithmetic mean and standard deviation (SD)). In case of an asymmetric distribution skewed to the right, e.g., with a relatively higher frequency of low values and a relatively lower frequency of high values, the median is lower than the arithmetic mean. Therefore, using the arithmetic mean in skewed data will tend to underestimate the prevalence of inadequate iron intake, and instead nonparametric statistics (median and percentiles) should be used [13]. In studies, where medians and percentiles are presented, these are quoted. In studies not presenting medians and percentiles, arithmetic means and SDs are quoted instead.

## 3. Results

Most reports were in English language, a few reports in other languages were translated into English. An overview of the 24 included European surveys/studies on dietary iron intake in pregnant women performed in 14 countries during the years 1991 to 2014 is shown in Table 1 [16–39]. The two Finnish reports [19, 20] contained the same sample of women but were analyzed using different dietary aspects and should be considered as being one study.

The age of the women ranged 18 to 42 years. In most studies the mean age was around 30 years.

The dietary survey methods varied between the 24 studies (Table 1). The most common dietary method was FFQ, being used in 9 studies; 3–7 day food diary being used 8 studies and 24-hour dietary recall performed 1–3 times being used in 6 studies. Four studies [19, 28, 31, 36] performed 3–5 day food diary concomitantly with an FFQ, and one study used 24-hour recall × 2 concomitantly with an FFQ [38].

The food composition tables being used to calculate dietary iron intake were in most countries based on national food databases, but a Greek [23] and one Spanish study [33] used food composition tables from USA and the second Spanish study used tables from France [32] in the calculation of micronutrient intake.

In Table 1, the studies are arranged in alphabetic order according to the country. In Table 2, where the FFQ studies are excluded, the studies are arranged according to the magnitude of median or mean dietary iron intake.

Among all the studies (FFQs plus Dietary Records), the Czech Republic, Finland, Germany, Greece, Portugal, and Spain (INMA-Valencia) reported a median or mean dietary iron intake ranging from 11.4 to 20.4 mg/day. In Norway, England, Croatia, and Poland, iron intake was approximately 10-11 mg/day, and in Bosnia and Spain (Reus), the intake was below 9 mg/day. In the studies using Dietary Records, the dietary iron intake in 11 countries varied between 8.1–15.4 mg/day as shown in Table 2; the estimated “median” value of dietary iron intake in the 11 countries was 10-11 mg/day. Dietary iron intake in nonpregnant women of reproductive age in the respective countries is shown as well [16, 40–45]. Clearly, the dietary iron intake in pregnant women did not differ significantly compared with the intake in nonpregnant women.

Five studies evaluated the results of FFQs against the results of food diary and 24-hour recall methods [19, 28, 31, 36, 38]. In all these studies, intake of nutrients and iron was significantly higher in the FFQ studies than in the Dietary Record studies with *p* values ranging from *p* < 0.01 [31, 36, 38] to *p* < 0.05 [28]. Median or mean values for dietary iron intake in mg/day in FFQ studies versus Dietary Record studies were 16.5 versus 12 [19], 11 versus 10 [28], 16.2 versus 14.3 [31], 15 versus 10.1 [36], and 11.2 versus 8 mg/day [39]. The correlations between iron intake in FFQ and Dietary Record studies were weak with crude Pearson correlation coefficients (*r*) ranging from 0.27–0.32 [19, 28, 36, 38] and 0.43 [31]; when adjusted for energy intake, the coefficients increased slightly to 0.41–0.56 [19, 28, 31, 36].

The discrepancy between FFQ and Dietary Record methods was clearly shown in the Swedish study [34]. The reference group of nonpregnant women (*n* = 206) had a dietary iron intake of mean 14.5 mg/day [34], which was significantly higher compared to a mean of 9.4 mg/day in the large Nationwide Swedish study using 4-day food diary [13, 46]. The authors concluded “that there was some uncertainty concerning the dietary records.” In Spain, the INMA-Valencia FFQ study reported a mean iron intake of 20.7 mg/day [33] in contrast to the 7-day food diary study from nearby Reus reporting a mean intake of approximately 8.3 mg/day [32].

The Croatian and Czech studies [17, 18] reported a significant increase in energy, macronutrient, and micronutrient intake including iron, during the three trimesters (*p* < 0.001). In contrast, the studies from Germany [22], Greece (Pireus) [23], Hungary [25], Portugal [31], Spain (Reus) [32], and England (London) [37] showed no statistically significant differences between nutrient and iron intake in the various trimesters.

Three studies [24, 26, 27] included the energy intake in the selection criteria of the women. In the Greek [24] and Norwegian studies [27], only women with a habitual energy intake between 4.5 and 20 MJ/day were included as suggested in an Australian study [47]. In the Irish study [26] under-reporters and over-reporters of energy intake, in total 122 out of 524 (23%) women, were excluded from the study.

Heme iron intake in percentage of total iron intake was reported in the Croatian study, being 15.8%, 16.4%, and 16.6% in the 1st, 2nd, and 3rd trimester, respectively [17]. In the study from Leeds, heme iron constituted 5.2% of dietary iron intake [39].

The nutrient density for iron was reported only in the Irish study, being median 17.0 mg per 10 MJ (mean 19.5 ± 0.8 (SD)) [26], which is among the highest nutrient densities reported in Europe [13].

The Norwegian MoBa study recorded the use of dietary supplements [27]. Among the women, 22% recorded the use of an iron supplement *per se* and a further 25% took supplemental iron contained in a multivitamin-multimineral tablet. Women using any kind of supplement had a significantly higher dietary iron intake than nonsupplement users (*p* < 0.001) (see Table 1).

The Average Requirement (AR) is the level of daily nutrient intake that is estimated to be adequate for half of the people in a population group, provided a normal distribution of requirement [48]. Two studies from Portugal [30] and Spain [33] quoted the AR, with an estimated value of 22 mg iron/day. Provided an AR of 22 mg/day for dietary iron intake in pregnant women, more than 60–80% of women in all the countries had an intake below AR, in some countries up to 100% (Table 1).

In the various studies, there was no consistency in the terminology and the use of Dietary Reference Values (DRV) for dietary iron intake [48]. Eight studies quoted the Recommended Dietary Allowance (RDA), 6 studies the Reference Nutrient Intake (RNI), 4 studies the Dietary Reference Intake (DRI), and two studies the AR.

The recommended intake of dietary iron in pregnant women by the national nutrition boards displayed major differences between countries, varying from 14 mg/day in Ireland [26] to 14.8 mg/day in UK [39], 15 mg in Norway [27] and Sweden [34], 18 mg/day in Spain [32], and 30 mg/day in Germany [23] (see Table 1).

Table 1 shows the Dietary Record studies, which assessed the percentage of women having an iron intake below the recommend intake. This fraction was dependent on the national recommended intake being hold against the actual intake and therefore varied between countries, with an overall range of 60–100%.

When median iron intake is below the recommended intake, this indicates that more than 50% of the women have an iron intake below the recommended intake. In all the Dietary Record studies, median iron intake was considerably lower than the recommended intake, indicating that more than 50% of the women had an inadequate iron intake.

## 4. Discussion

In the reported Dietary Record studies, dietary iron intake was distinctly below the recommend intake in 60–100% of pregnant women. This finding was evident even in countries (Finland, Norway, Sweden, and England), which recommend the same iron intake in pregnant as in nonpregnant women of reproductive age. One exception was the Irish FFQ study, which reported a median dietary iron intake of 17.0 mg/day (mean 19.3 mg/day), indicating that approximately 37% had an intake below the RDA of 14 mg/day [26]. However, this was an FFQ study, which excluded a considerable number of women with nonplausible energy intake, and the RDA for iron was the lowest recommended value reported among the European countries in this paper. For comparison, mean dietary iron intake in women of reproductive age in Ireland assessed by Dietary Records was 10.1 mg/day in the NSIFCS study and 13.7 mg/day in the NANS study [49], clearly being significantly lower than the reported intake in pregnant women [26].

The exclusion of women with nonplausible low energy intake pushes the population median and mean intake upwards but might be necessary to get a “true” picture of what intake is in this population. Energy intake criteria for inclusion were also used in the Greek [24] and Norwegian [27] studies.

The DRI value of 30 mg/day used in one Greek study [23] is due to a quotation error and should instead be 27 mg/day. The authors state that this value is recommend by the Institute of Medicine (IOM) [50], but actually it has been taken from a position paper from the American Dietary Association [51], which does not quote the IOM but quotes the recommendation from the Centers for Disease Control and Prevention (CDC) stating that all pregnant women should take an iron supplement of 30 mg/day during pregnancy [52].

The studies covered a time period of more than 25 years, and it appears that the overall dietary iron intake was quite constant during this period.

Four studies [23, 25, 31, 32] reported that dietary iron intake was constant during the three trimesters of pregnancy, indicating that most women do not to any significant extent change their dietary habits during pregnancy. In contrast, the Croatian [17] and Czech [18] studies reported a significant increase in energy, macro- and micronutrient intake including iron during pregnancy, probably reflecting country specific dietary habits and/or recommendations from the antenatal health authorities.

In the various countries, dietary iron intake was quite similar in pregnant and nonpregnant women of reproductive age (Table 2). The studies from Reus [32] and London [37] showed that iron intake was not significantly different prior to pregnancy, during gestation, and in the postpartum period. This indicates that most women do not to any significant extent change their dietary habits when they become pregnant but continue with their habitual prepregnancy diet during pregnancy.

The various designs of the studies and the different dietary methods impede direct comparison of the results. Clearly, when comparing FFQs and Dietary Records, dietary iron intake was significantly higher in FFQs than in Dietary Records and with low correlation coefficients (see above). This finding has previously been reported in nonpregnant women of reproductive age in Europe [13]. However, our findings are in contrast with the conclusions of a previous review paper, which evaluated different dietary methods for assessment of micronutrient intake in pregnant women, and concluded that “FFQs were good for measuring both short-term and long-term intake of iron” [53].

The European Food Consumption Survey Method (EFCOSUM) group has concluded that “the most suitable method to get internationally comparable new data on population means and distributions of actual intake is a 24-hour recall, to be conducted at least twice” [54]. However, this method was used in only 6 studies.

National food composition tables reflect the composition of the most common staple foods consumed in a country. Usually, mandatory fortification of foods is included in the food composition tables, while optional fortification is not. There may in some countries exist foods which are iron-fortified on a voluntary basis, and this iron will not be included in the food composition tables. Iron fortified foods will contribute to a higher iron nutrient density and consequently to a higher dietary iron intake [13]. This could in part explain the differences in dietary iron intake across Europe.

Countries have different recommendations concerning iron fortification of foods. For example, UK has mandatory fortification of wheat flour with iron. Many breakfast cereals are fortified with iron on an optional basis and according to the British National Diet and Nutrition Survey contribute to 20% of the average iron intake in British adults. Fortification practices in Europe in 2006 were as follows: Denmark, Finland, Germany, Ireland, Italy, the Netherlands, and Spain had no mandatory iron fortification, but optional fortification with iron is occasionally used, especially in flour and breakfast cereals [55].

In the Norwegian MoBa study [27], users of any dietary supplement had a higher dietary iron intake than nonusers, probably due to more healthy dietary habits, because supplement users had a higher educational level, a lower frequency of smoking, and a higher frequency of normal body weight prior to pregnancy.

The frequency distribution of dietary iron intake in a population does not show a normal distribution, but a distribution, which is skewed to the right [13], with an overweight of high values. Therefore, median values are consistently lower than arithmetic values as shown in four studies [26, 27, 33, 35]. The distribution is therefore most accurately described using nonparametric statistics (median and percentiles) or using logarithmic values in the calculation of the geometric mean and standard deviation [13]. Using the arithmetic mean will underestimate the frequency of individuals with an inadequate iron intake.

National and international recommendations for dietary iron intake are shown in Table 3.

RNI and AR for dietary iron in pregnant women are compared with RNI and AR for nonpregnant women of reproductive age in the respective countries [50, 56–60]. Obviously, there is no consensus on the recommended intake. The IOM [50] advocates for an increased dietary iron intake in pregnancy, while the other institutions [56–60] recommend the same intake in pregnant and nonpregnant women.

However, the CDC [52], the Food and Agricultural Organization of the United Nations (FAO), and WHO [56] as well as the Nordic Nutrition Recommendations (NNR) [57] conclude that an adequate iron intake cannot be obtained solely by changes in dietary habits and therefore recommend iron supplements during pregnancy. In contrast, the European Food Safety Authority (EFSA) [58, 59] and the Scientific Advisory Committee on Nutrition (SACN) [60] do not recommend supplementary iron to pregnant women as routine prophylaxis but argue that treatment with iron should be reserved to women with confirmed ID or IDA.

In nonpregnant Chinese women of reproductive age, the average physiological requirements for absorbed iron measured by a stable iron isotope method and using linear regression is approximately 1.29 mg/day (20.98 μg/kg body weight/day after adjustment for body mass) [61]. The calculated AR for iron is approximately 11 to 13 mg/day and the RNI is between 15 to 18 mg/day [61].

The physiological need for absorbed iron during normal pregnancy is substantial and increases gradually during gestation from approximately 1 mg/day in the 1st trimester to 7.5 mg/day in the 3rd trimester [5]. The extra iron is needed in order to expand the woman's red cell mass and to secure an adequate iron supply for a normal function of the placenta and the development of the growing fetus. The total gross need for iron in a normal pregnancy is 1,000–1,200 mg with a net need of approximately 500–600 mg [5]. Women with prepregnancy body iron reserves of approximately 500 mg corresponding to a serum ferritin level of 60–70 *μ*g/L will be able to go through a normal pregnancy without taking iron supplements and without getting ID or IDA [14]. Among Danish women with prepregnancy ferritin levels below 60–70 *μ*g/L, who are not taking iron supplements, approximately 75%, develop ID and 20–25% IDA [62].

The study from Leeds found a positive relationship between the total iron intake (dietary plus supplemental iron) in early pregnancy and the birthweight of the newborns [39].

In the assessment of iron status in pregnant women, it is important to distinguish between iron-supplemented and nonsupplemented women. An overview of iron status studies in pregnant women in Europe who were not taking iron supplements have recently been published [12]; median serum ferritin levels in these studies ranged 5–21 *μ*g/L (“estimated” median value 10 *μ*g/L); the frequency of ID ranged 35–83% (“estimated” median value 50%) and the frequency of IDA ranged 12–49% (“estimated” median value 26%). In women not taking iron supplements, the frequency of ID and IDA typically increases gradually during pregnancy and peaks in mid or late 3rd trimester [62].

In contrast, iron-supplemented pregnant women had a higher iron status than nonsupplemented women; median serum ferritin levels in the iron supplemented women ranged 15–63 *μ*g/L (“estimated” median value 21 *μ*g/L); the frequency of ID ranged 0–41% (“estimated” median value 4%) and the frequency of IDA ranged 0–27% (“estimated” median value 5%) [12]. Thus, the prevalence of ID and IDA was markedly lower in iron-supplemented women compared with nonsupplemented women [12].

The low dietary iron intake in pregnant women has motivated the CDC [52], FAO & WHO [56], and NNR [57] guidelines to recommend routine iron prophylaxis during pregnancy. In Denmark, all pregnant women are recommended a supplement of 40–50 mg elemental iron/day at their first visit to the antenatal clinic and the compliance is high, close to 80% [63].

From a physiological point of view, individual iron prophylaxis should be encouraged instead of general routine prophylaxis [14], because 20–30% of the pregnant women in fact do not need iron supplements and consequently are “overtreated.” Routine evaluation of iron status (serum ferritin and serum transferrin saturation) in pregnant women at their first check-up in the antenatal clinic can identify women who should be prescribed iron supplements [14]. This approach is recommended by the Danish Society of Obstetrics and Gynecology [64] but has still not been implemented by the Danish Health Authority.

### 4.1. Limitations of This Review

This review has limitations, mainly due to the heterogeneous methods used in the studies. All but one study [27] were regional and do not reflect the nutritional situation in the entire country. The dietary methods differed between studies, statistical methods were different, most studies used parametric and a few nonparametric statistics, few studies had been corrected for under- and over-reporting, and furthermore, there was an inconsistent terminology concerning the use of DRVs of dietary iron. These are all factors, which impair comparison of the results of the various studies. Furthermore, the food composition tables varied between countries, and the contribution of mandatory and/or voluntary food iron fortification was not evaluated in the studies.

## 5. Conclusions

This review demonstrates that in Europe, most women do not change their dietary habits during pregnancy and women consume the same amounts of micronutrients before, during, and after pregnancy. It is important to recognize that a high proportion of pregnant women, in several studies 80–100%, has a dietary iron intake, which is below the recommended intake. The relatively low iron intake contributes to the low body iron status found in many pregnant women not taking iron supplements [12, 62]. This has motivated several advisory institutions to recommend routine iron supplementation during pregnancy [52, 56, 57]. However, few European countries follow these recommendations. In Denmark, the National Health Authority has successfully implemented this recommendation as a mandatory procedure in the antenatal health care system [65].

In European countries and within the European Union, there is a need to obtain consensus between the various guidelines and the conflicting issue of iron supplementation. Furthermore, there is a need for implementation of common standardized Dietary Record methods [54] and for standardization of food composition tables as initiated by EFSA [66]. It is also important to reach consensus on the use of the different DRVs [48] and to implement the use of uniform statistical methods in order to perform more reliable intra- and intercountry comparison of dietary intake.

## Figures and Tables

**Table 1:** Dietary iron intake in pregnant women in European countries.

Country (city or region)	Study period	Women (n)	Age (years) mean ± SD and/or range	Trimester	Dietary method	Dietary iron mg/day			Recommended iron intake at time of study mg/day	Iron intake below recommended % of women	Reference
						Mean ± SD^2^	Median	IQ^3^ range, CI^4^, percentiles^5^			
Bosnia (Canton Una Sana)	∼2014	∼40	25.6 ± 1.7	Not stated	24-hour recall	8.6			RDA 27	100% had intake <RDA	[16]
Croatia (Osijek)	2010–2011	222	Not stated	1st	24-hour recall		9.5	7.5–12.4^3^	DRI 27	100% had intake <DRI	[17]
Croatia (Osijek)	2010–2011	222	Not stated	2nd	24-hour recall		10.1	7.8–13.3^3^	DRI 27	100% had intake <DRI	[17]
Croatia (Osijek)	2010–2011	222	Not stated	3rd	24-hour recall		11.2	8.7–14.6	DRI 27	100% had intake <DRI	[17]
Czech Republic (Hradec Kralove Region)	2009–2010	152	28.9 ± 3.6	1st	7-day food diary	14.0 ± 3.5			RDA 27	52% had intake <RDA	[18]
Czech Republic (Hradec Kralove Region)	2009–2010	152	∼28.9 ± 3.6	2nd	7-day food diary	15.3 ± 3.5			RDA 27	57% had intake <RDA	[18]
Czech Republic (Hradec Kralove Region)	2009–2010	152	∼28.9 ± 3.6	3rd	7-day food diary	16.3 ± 3.5			RDA 27	60% had intake <RDA	[18]
Finland (Oulu)	1995–1996	113	29.6 ± 5.1	3rd	FFQ^1^	16.5 ± 4.5			RDA 15^7^		[19]
Finland (Oulu)	1995–1996	113	29.6 ± 5.1	3rd	5-day food diary × 2	12 ± 4			RDA 15^7^		[19]
Finland (Oulu)	1995–1996	118	29.6 ± 5.1	3rd	5-day food diary × 2		11.4	9.6–12.9^3^	RDA 15^7^		[20]
Finland (Oulu & Tampere)	1998–1999	797	29.6 ± 5.1	1st & 2nd & 3rd	FFQ^1^	18 ± 6			RDA 15^7^		[21]
Germany (Hamburg)	2011–2013	200 (95% caucasian)	31 ± 3.5	1st	24-hour recall	12 ± 4			RDA 30	100% had intake <RDA	[22]
Germany (Hamburg)	2011–2013	200 (95% caucasian)	∼31 ± 3.5	2nd	24-hour recall	13 ± 3			RDA 30	100% had intake <RDA	[22]
Germany (Hamburg)	2011–2013	200 (95% caucasian)	∼31 ± 3.5	3rd	24-hour recall	12 ± 3			RDA 30	100% had intake <RDA	[22]
Germany (Hamburg)	2011–2013	200 (95% caucasian)	∼31 ± 3.5	1st & 2nd & 3rd	24-hour recall	12 ± 2			RDA 30	100% had intake <RDA	[22]
Greece (Pireus)	2003	98	18–40	2nd	FFQ	11.6 ± 5.0		10.6–12.6^4^	DRI 30 (27)^see text^	100% had intake <DRI	[23]
Greece (Pireus)	2003	102	18–40	3rd	FFQ	11.9 ± 4.9		11.0–12.9^4^	DRI 30 (27)^see text^	100% had intake <DRI	[23]
Greece (Athens)	2012	56	18–42	1st & 2nd	24-hour recall × 3	∼15.4 ± 6.4			DRI 27	98% had intake <DRI	[24]
Hungary (Budapest)	1990–1994	70	22.9 ± 4.4 (19–38)	1st, 2nd & 3rd	FFQ	11.3 ± 4.1			RDA 20	81% had intake <70% of RDA	[25]
Ireland (Dublin)	2013	402	30.8 ± 5.2	1st	FFQ	19.3 ± 10.3	17.0		RDA 14	37% had intake <RDA	[26]
Norway (Nationwide MoBa study)	2002–2005	7,455 nonsupplement users	29.2 ± 4.8	2nd	FFQ	10.8 ± 3.4	10.3	6.1–17.1^5^	RDA 15^7^		[27]
Norway (Nationwide MoBa Study)	2002–2005	32,653 supplement users	29.2 ± 4.8	2nd	FFQ	11.3 ± 3.4	10.9	6.6–17.6^5^	RDA 15^7^		[27]
Norway (Oslo Region)	2003–2004	119	31.2 ± 4.1 (23–44)	2nd	FFQ		11	7–19^5^	RDA 15^7^		[28]
Norway (Oslo Region)	2003–2004	119	31.2 ± 4.1 (23–44)	2nd	4-day weighed food diary		10	7–15^5^	RDA 15^7^		[28]
Poland (Nationwide)	2009	512	20–35	3rd	7-day food diary	10.1			RDA 24	∼100% had intake <RDA	[29]
Portugal (Porto)	2004–2005	249	29.2 ± 6.6 (18–40)	3rd	FFQ		16.0	12.5–19.2^3^	AR 22	88% had intake <AR	[30]
Portugal (Porto)	2004–2005	70	∼29.8 ± 4.9	3rd	FFQ × 2	16.2 ± 5.5					[31]
Portugal (Porto)	2004–2005	101	29.8 ± 4.9	1st	3-day food diary	14.4 ± 4.8					[31]
Portugal (Porto)	2004–2005	101	∼29.8 ± 4.9	2nd	3 days food diary	14.3 ± 4.9					[31]
Portugal (Porto)	2004–2005	101	∼29.8 ± 4.9	3rd	3-day food diary	14.3 ± 5.0					[31]
Portugal (Porto)	2004–2005	101	∼29.8 ± 4.9	1st & 2nd & 3rd	3-day food diary	14.4 ± 3.7					[31]
Spain (Reus)	1991–1995	80	24–35	Preconception ≤12 weeks	7-day food diary	8.3 ± 3.0			RDA 18	∼98% had intake <RDA	[32]
Spain (Reus)	1991–1995	80	24–35	1st	7-day food diary	∼8.1 ± 2.1			RDA 18	∼98% had intake <RDA	[32]
Spain (Reus)	1991–1995	80	24–35	3rd	7-day food diary	∼8.5 ± 2.8			RDA 18	∼98% had intake <RDA	[32]
Spain (Reus)	1991–1995	80	24–35	Postpartum 6 weeks	7-day food diary	8.2 ± 2.3			RDA 18	∼98% had intake <RDA	[32]
Spain (INMA-Valencia)	2004–2005	822	∼17–40	1st	FFQ	20.7 ± 3.4	20.4	15.6–27.0^5^	AR 22 DRI 27	68% had intake <DRI	[33]
Sweden (Umeå)^∗^	2006–2009	209	23–40	1st	FFQ	∼13.1		∼12.5–13.8^4^	RDA 15^7^	98% had intake <RDA	[34]
UK (south West England) (ALSPAC)	1991–1992	11,923	27.9 ± 5.0 (15–44)	3rd	FFQ	10.4 ± 3.3	10.2	5.4–16.2^5^	RNI 14.8	∼75% had intake <RNI	[35]
UK (Southampton)	1991–1992	569 caucasians	26.4 ± 4.8	1st	FFQ		15.0	12.2–18.6^3^	RNI 14.8		[36]
UK (Southampton)	1991–1992	569 causasians	26.4 ± 4.8	1st	4-day food diary		10.1	7.9–12.4^3^	RNI 14.8		[36]
UK (London)	2002	42 caucasians	33.2 ± 4.6 (19–40)	1st	4–7 day weighed food diary	12.3 ± 4.6			RNI 14.8	71% had intake <RNI	[37]
UK (London)	2002	42 caucasians	∼33.2 ± 4.6 (19–40)	2nd	4–7 day weighed food diary	12.1 ± 3.5			RNI 14.8	79% had intake <RNI	[37]
UK (London)	2002	42 caucasians	∼33.2 ± 4.6 (19–40)	3rd	4–7 day weighed food diary	13.0 ± 5.3			RNI 14.8	67% had intake <RNI	[37]
UK (London)	2002	42 caucasians	∼33.2 ± 4.6 (19–40)	Postpartum	4–7 day weighed food diary	12.3 ± 4.5			RNI 14.8	81% had intake <RNI	[37]
UK (Sheffield)	2003–2004	123 caucasians	29 ± 6.4 (17–43)	1st	FFQ	11.2 ± 3.9		4.7–21.1^6^	RNI 14.8		[38]
UK (Sheffield)	2003–2004	123 caucasians	29 ± 6.4 (17–43)	1st	24-hour recall × 2	8.0 ± 2.8		2.6–17.8^6^	RNI 14.8		[38]
UK (Leeds)	2003–2006	1,257	18–45	1st	24-hour recall	11.5 ± 5.3			RNI 14.8	80% had intake <RNI	[39]

^1^FFQ = Food Frequency Questionnaire; ^2^SD = standard deviation; ^3^IQ range = interquartile range, 25–75 percentiles; ^4^CI = 95% confidence interval; ^5^5–95 percentile range; ^6^observed range; ^7^RDA for women of reproductive age. No specific recommendation for pregnant women; they are recommended oral iron supplements. AR = Estimated Average Requirement; daily intake levels estimated to meet the needs of half of the healthy pregnant women. DRI = Dietary Reference Intake; nutrition recommendations from the Institute of Medicine of the National Academies (USA). RDA = Recommended Dietary Allowance; daily intake adequate to meet the nutrient requirements of nearly all (97%–98%) healthy pregnant women. RNI = Reference Nutrient Intake; daily intake adequate to meet the nutrient requirements of nearly all (97.5%) healthy pregnant women.

**Table 2:** Dietary iron intake in pregnant women in 11 European countries arranged according to the magnitude of median or mean iron intake. For comparison, iron intake in nonpregnant women of reproductive age in the same countries is shown as well. Only studies using dietary record methods are included.

Country	Median or mean^*∗*^ dietary iron intake in pregnant women mg/day	Reference	Median or mean^*∗*^ dietary iron intake in nonpregnant women mg/day	Reference
Greece	15.4^*a*+*b*^^*∗*^	[24]		
Czech Republic	14.0^*a*^^*∗*^; 15.3^*b*^^*∗*^; 16.3^*c*^^*∗*^	[18]		
Portugal	14.4^*a*+*b*+*c*^^*∗*^	[31]		
Germany	12.2^*a*+*b*+*c*^^*∗*^	[22]	12.2	[40]
Finland	11.4^*c*^	[20]	10.3	[41]
Norway	11^*b*^^*∗*^; 11^*c*^^*∗*^; 10^*b*^	[28]	10.0	[42]
England	10.1^*a*^; 8.0^*a*^^*∗*^; 11.5^*a*^^*∗*^	[36, 38, 39]	9.5	[43]
Croatia	9.5^*a*^; 10.1^*b*^; 11.2^c^	[17]		
Poland	10.1^*c*^^*∗*^	[29]	10.7^*∗*^	[44]
Bosnia	8.6^*∗*^	[16]	7.6^*∗*^	[16]
Spain	8.3^*a*^^*∗*^; 8.5^*c*^^*∗*^	[32]	10.5^*∗*^	[45]

^*∗*^Arithmetic mean, *a* = 1st trimester; *b* = 2nd trimester; *c* = 3rd trimester.

**Table 3:** Dietary Reference Values for iron. Reference Nutrient Intake and Average Requirement for dietary iron in pregnant women compared with nonpregnant women of reproductive age.

Institution	DRV for iron in pregnant women mg/day		During pregnancy	DRV for iron in nonpregnant women mg/day		Reference
	RNI	AR		RNI	AR	
IOM 2001	27	22		18	5	[50]
FAO &WHO 2001	19.6^*∗*^		Iron supplement recommended	19.6^*∗*^		[56]
NNR 2012	15	6	Iron supplement recommended	15	6	[57]
EFSA 2015	16	6		16	6	[58, 59]
SACN 2017	14.8	11.4		14.8	11.4	[60]

^*∗*^Provided 15% bioavailability of dietary iron. DRV = Dietary Reference Value. RNI = Reference Nutrient Intake. AR = Average Requirement. IOM = Institute of Medicine (USA). FAO = Food and Agriculture Organization of the United Nations. WHO = World Health Organization. NNR = Nordic Nutrition Recommendations. EFSA = European Food Safety Authority. SACN = Scientific Advisory Committee on Nutrition (UK).
